# Landslide Susceptibility Mapping Using Machine Learning Algorithms and Remote Sensing Data in a Tropical Environment

**DOI:** 10.3390/ijerph17144933

**Published:** 2020-07-08

**Authors:** Viet-Ha Nhu, Ayub Mohammadi, Himan Shahabi, Baharin Bin Ahmad, Nadhir Al-Ansari, Ataollah Shirzadi, John J. Clague, Abolfazl Jaafari, Wei Chen, Hoang Nguyen

**Affiliations:** 1 Geographic Information Science Research Group, Ton Duc Thang University, Ho Chi Minh City 700000, Vietnam; nhuvietha@tdtu.edu.vn; 2 Faculty of Environment and Labour Safety, Ton Duc Thang University, Ho Chi Minh City 700000, Vietnam; 3 Department of Remote Sensing and GIS, University of Tabriz, Tabriz 51666-16471, Iran; mohammadi.ayub@tabrizu.ac.ir; 4 Department of Geomorphology, Faculty of Natural Resources, University of Kurdistan, Sanandaj 66177-15175, Iran; 5 Board Member of Department of Zrebar Lake Environmental Research, Kurdistan Studies Institute, University of Kurdistan, Sanandaj 66177-15175, Iran; 6 Faculty of Built Environment and Surveying, Universiti Teknologi Malaysia (UTM), Johor Bahru 81310, Malaysia; baharinahmad@utm.my; 7 Department of Civil, Environmental and Natural Resources Engineering, Lulea University of Technology, 971 87 Lulea, Sweden; 8 Department of Rangeland and Watershed Management, Faculty of Natural Resources, University of Kurdistan, Sanandaj 66177-15175, Iran; a.shirzadi@uok.ac.ir; 9 Department of Earth Sciences, Simon Fraser University, 8888 University Drive, Burnaby, BC V5A 1S6, Canada; jclague@sfu.ca; 10 Research Institute of Forests and Rangelands, Agricultural Research, Education, and Extension Organization (AREEO), Tehran P.O. Box 64414-356, Iran; jaafari@rifr-ac.ir; 11 College of Geology & Environment, Xi’an University of Science and Technology, Xi’an 710054, China; chenwei0930@xust.edu.cn; 12 Key Laboratory of Coal Resources Exploration and Comprehensive Utilization, Ministry of Natural Resources, Xi’an 710021, Shaanxi, China; 13 Institute of Research and Development, Duy Tan University, Da Nang 550000, Vietnam; nguyenhoang23@duytan.edu.vn

**Keywords:** machine learning, AdaBoost, alternating decision tree, ensemble model, Cameron Highlands, Malaysia

## Abstract

We used AdaBoost (AB), alternating decision tree (ADTree), and their combination as an ensemble model (AB-ADTree) to spatially predict landslides in the Cameron Highlands, Malaysia. The models were trained with a database of 152 landslides compiled using Synthetic Aperture Radar Interferometry, Google Earth images, and field surveys, and 17 conditioning factors (slope, aspect, elevation, distance to road, distance to river, proximity to fault, road density, river density, normalized difference vegetation index, rainfall, land cover, lithology, soil types, curvature, profile curvature, stream power index, and topographic wetness index). We carried out the validation process using the area under the receiver operating characteristic curve (AUC) and several parametric and non-parametric performance metrics, including positive predictive value, negative predictive value, sensitivity, specificity, accuracy, root mean square error, and the Friedman and Wilcoxon sign rank tests. The AB model (AUC = 0.96) performed better than the ensemble AB-ADTree model (AUC = 0.94) and successfully outperformed the ADTree model (AUC = 0.59) in predicting landslide susceptibility. Our findings provide insights into the development of more efficient and accurate landslide predictive models that can be used by decision makers and land-use managers to mitigate landslide hazards.

## 1. Introduction

Landslides are the slow to rapid downslope movement of Earth materials triggered by a wide variety of natural processes, as well as by land surface disturbances due to human activities [1,2,3,4,5,6]. Whether triggered naturally or by human activities, landslides are responsible for much economic damage and loss of life each year [7,8,9]. Notably, recurrent landslides along roads and on other cut slopes in mountainous regions pose a great threat to the people living in these areas [3,5,10,11,12].

In Malaysia, landslides pose a constant threat to infrastructure, agriculture, other natural resources, and tourism, and local and central governments are strained financially and logistically in dealing with them [8,13]. Most landslides in Malaysia are triggered by heavy rainfall [9,13]. One area in the country that is particularly impacted by landslides, due in part to increased urbanization and expanded plantation agriculture, is the Cameron Highlands in the central part of peninsular Malaysia [6,8].

During the past two decades, major advances in computing power, remote sensing, and geographic information systems have facilitated the preparation of landslide susceptibility maps. These maps can be used by policy and decision makers to mitigate social and economic losses from landslides. A wide variety of models and methods for landslide susceptibility mapping have been proposed over this period, including: (1) knowledge-based approaches such as analytical hierarchy process [14]; (2) empirical approaches (statistical bivariate and multivariate methods) [15] such as frequency ratio [16], certainty factor [17], index of entropy [18], geographically weighted principal component analysis [2], regression analysis [19], and the conditional analysis method [20]; and (3) machine learning methods such as artificial neural network (ANN) [21], support vector machine (SVM) [22], adaptive neuro fuzzy inference system (ANFIS) [23], decision trees [24,25,26,27], support vector regression (SVR) [28], and deep learning neural networks [29].

Given the complexity of landslide prediction, many researchers have turned their attention to using hybrid ensemble approaches that combine machine learning methods with metaheuristic algorithms [30,31] or ensemble learning techniques [32,33]. Examples of recent proposed and hybrid ensemble models for predicting landslides include ANN, SVM, SVR, and ANFIS integrated with genetic algorithm, particle swarm optimization, and gray wolf optimizer [30,34,35,36]; alternative decision tree (ADTree) combined with ensemble techniques such as AdaBoost (AB), Random Subspace, MultiBoost, Bagging, and Dagging [24,37]; SVM combined with ensemble techniques [38,39], Naïve Bayes tree coupled with Random Subspace [40], radial basis function ANN combined with Rotation Forest [41], best first decision tree combined with Rotation Forest [42], and Bayesian logistic regression combined with AB, MultiBoost, and Bagging [43]. Hybrid ensemble models also have been successfully used in studies of other hazards, including flooding [44,45,46,47,48], wildfire [34,49], sinkhole formation [50], dust storm [51], drought [52], gully erosion [53,54,55], and land subsidence [56], as well as in other environmental studies, such as land-use planning [57] and groundwater potential mapping [54,58,59,60,61,62].

This study applies AB, ADTree, and their combination in an ensemble model (AB-ADTree) to spatially predict landslide susceptibility in a part of the Cameron Highlands. Our paper provides insights into the development of more efficient and accurate landslide predictive models to aid decision makers and land-use managers in mitigating landslide hazards. The modeling process and visualization of landslide susceptibility maps were hosted in WEKA 3.7.12 and ArcGIS 10.2, respectively.

## 2. Study Area

**Figure 1:**
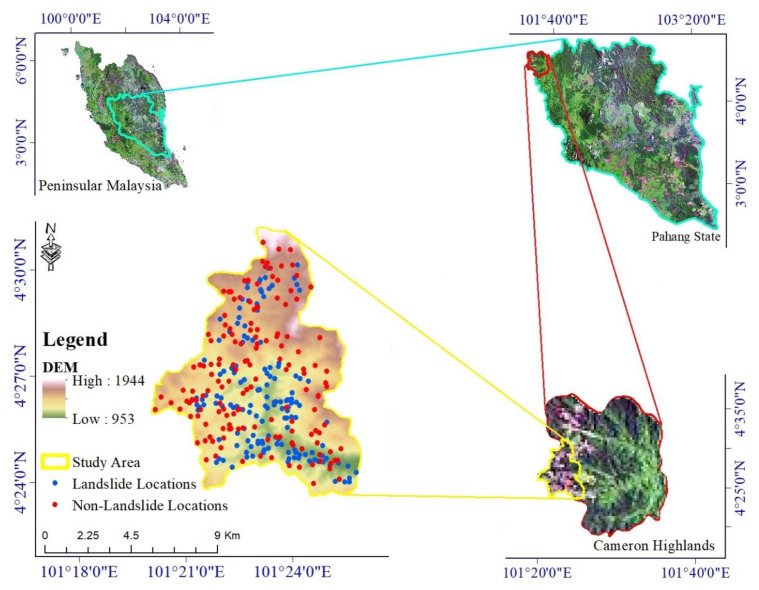
Geographical location of the study area and landslide and non-landslide locations used in the study.

The study area covers approximately 81 km^2^ of the southwestern Cameron Highlands and ranges from 953 to 1944 m above sea level [63] (Figure 1). The area is undergoing rapid land clearing that has exacerbated erosion and landslides [9]. Felsic intrusive rocks underlie most of the study area (61 km^2^), but Silurian–Ordovician metamorphic rocks (schist, phyllite, and slate) and minor sandstone and limestone are also present [6].

Malaysia is a tropical country that experiences heavy precipitation throughout the year [8]. About 3800–4200 mm of rainfall were recorded by the Tropical Rainfall Measuring Mission (TRMM) sensor in the study area in 2017 [6]. The country experiences wet seasons from September to December and from February to May. Peak rainfall in the Cameron Highlands occurs from March to May and from November to December. During these periods, rivers overflow their banks, causing extensive flooding.

## 3. Methodology

The first step in this study was to detect historical landslide locations and to identify a set of landslide conditioning factors. Using the InSAR technique and Google Earth images, and conducting multiple field surveys, we detected 152 landslides in the study area. In order to generate the training and validation datasets required for the modeling process, we randomly divided the landslide locations into two subsets: 122 landslides (80%) were selected for model training, and 30 landslides (20%) were used for model validation (20%). Since our modeling approach is based on a binary classification in which we develop a predictive model to distinguish between landslides and non-landslides, we randomly sampled 152 non-landslide locations in the study area (Figure 1). The end result is training and validation datasets that comprise, respectively, 244 and 60 samples.

We selected 17 landslide conditioning factors for this study based on the landslide literature, expert knowledge, and general characteristics of the study area. We developed three machine learning models (i.e., AB, ADTree, and AB-ADTree) to perform the landslide susceptibility mapping. The results were compared and validated using the Receiver Operating Characteristics (ROC) curve, statistical measurements, and the Friedman and Wilcoxon methods. The next subsections describe the steps in the research methodology in more detail.

### 3.1. Data Collection

**Figure 2:**
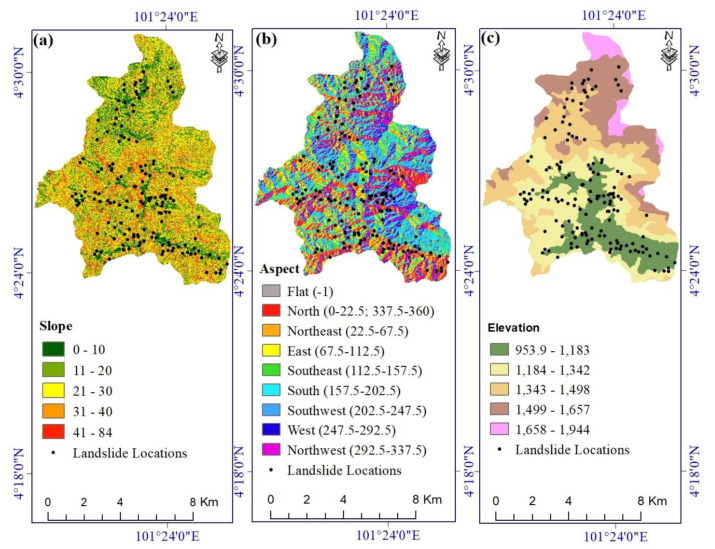
Landslide conditioning factors: (**a**) slope, (**b**) aspect, and (**c**) elevation.

**Figure 3:**
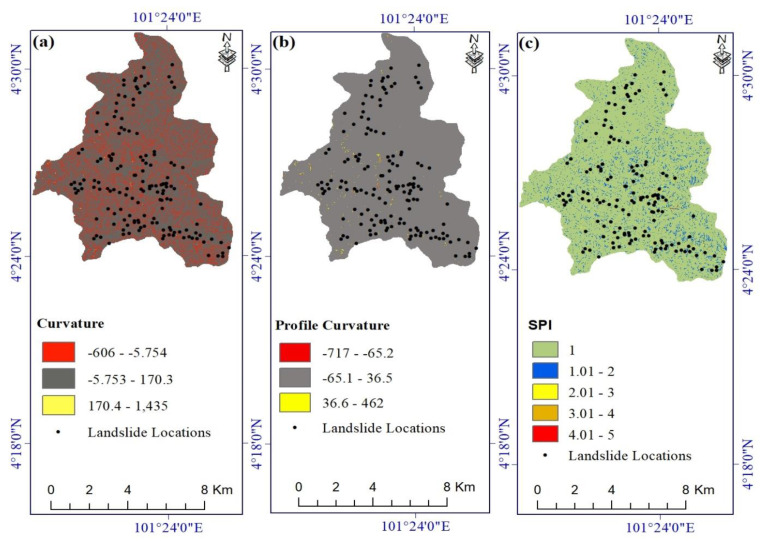
Landslide conditioning factors: (**a**) curvature, (**b**) profile curvature, and (**c**) SPI.

**Figure 4:**
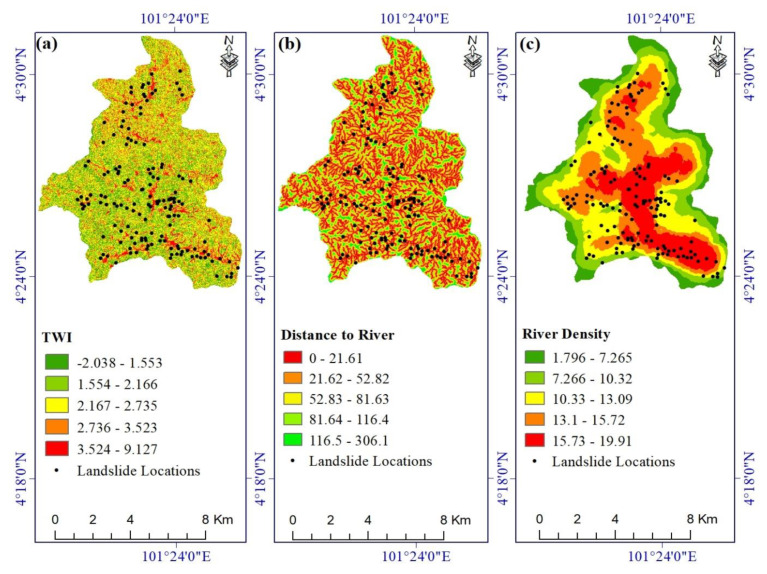
Landslide conditioning factors: (**a**) TWI, (**b**) distance to river, and (**c**) river density.

Table 1 lists the landslide conditioning factors used in this study, together with their sources and scales. We produced a 10 m resolution Digital Elevation Model (DEM) from Sentinel-1 satellite imagery acquired on 20 February 2017 and 2 March 2017, with a perpendicular baseline of 97 m. The DEM was created using an InSAR technique and Sentinel Application Platform (SNAP) software. Geographic Information System (GIS) layers extracted from the DEM include slope, aspect, elevation (Figure 2a–c); curvature, profile curvature, Stream Power Index (SPI) (Figure 3a–c); and Topographic Wetness Index (TWI) (Figure 4a). Rivers and streams were mapped on the DEM using the hydrology toolbox in ArcGIS, and that map was used to create the distance-to-river and river density layers (Figure 4b,c).

**Figure 5:**
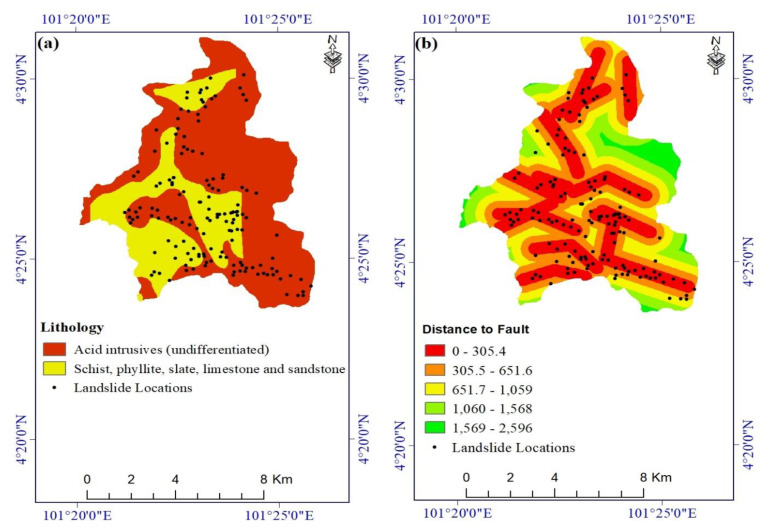
Landslide conditioning factors: (**a**) lithology and (**b**) distance to fault.

Unconsolidated sediments are prone to shallow slope failures because of their low cohesion and relatively high porosity, which leads to rapid water infiltration [64]. Bedrock near faults is commonly highly fractured and weathered, and thus it has much lower strength than non-faulted rock [65,66]. Accordingly, we digitized lithology and faults from a 1:100,000-scale geologic map acquired from the Malaysia Mineral and Geoscience Department (Figure 5a,b).

**Figure 6:**
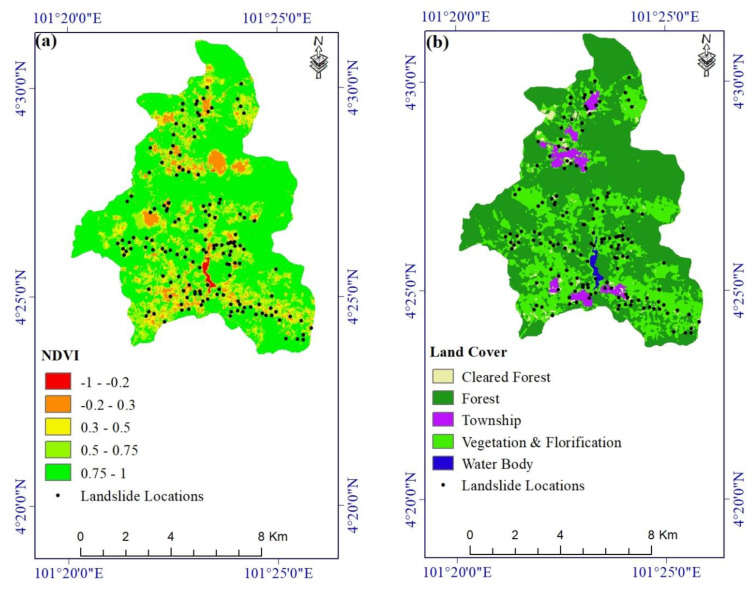
Landslide conditioning factors: (**a**) NDVI and (**b**) land cover.

Vegetation absorbs soil moisture and reduces erosion, and plant roots increase soil strength and may reduce the incidence of landslides [67]. Thus, slope failures are generally less common in areas with dense vegetation than in sparsely vegetated areas or on bare ground [68]. A map layer of the Normalized Difference Vegetation Index (NDVI) was created from Sentinel-2 satellite imagery acquired on 11 October 2017 using the formula Float (NIR − Red)/(NIR + Red). High amounts of chlorophyll result in low reflectance in the red band and high reflectance in the near-infrared band [69,70,71]. A high NDVI value indicates green vegetation, whereas a low value indicates sparse vegetation or bare ground [67] (Figure 6a).

A land-use map was extracted using Sentinel-1 and Landsat-8 images downloaded from the Copernicus and US Geological Survey websites (scihub.copernicus.eu and earthexplorer.usgs.gov). Five land-cover classes (forest, cleared forest, florification, water bodies, and township) were mapped and used for landslide susceptibility zonation (Figure 6b).

**Figure 7:**
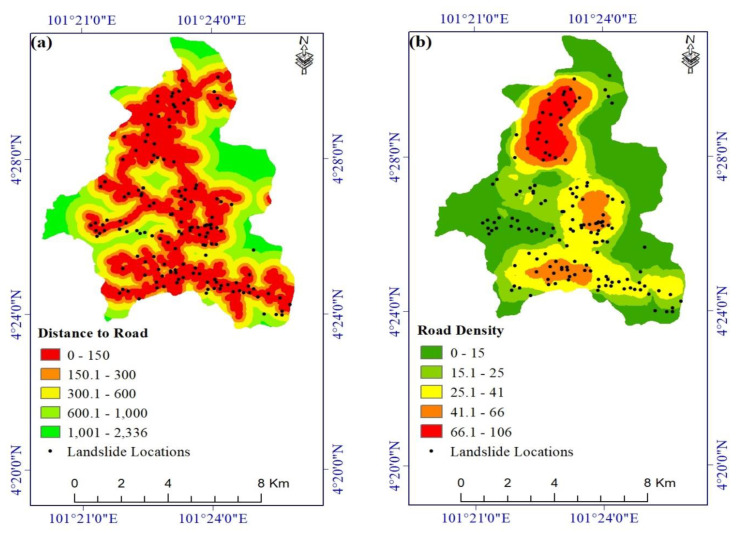
Landslide conditioning factors: (**a**) distance to road and (**b**) road density.

Roads are common locations of landslides, especially in mountainous areas [8,72]. The 32 km-long road network in the study area was taken from the Open Street Map. This layer was used to create distance-to-road and road density layers (Figure 7a,b).

**Figure 8:**
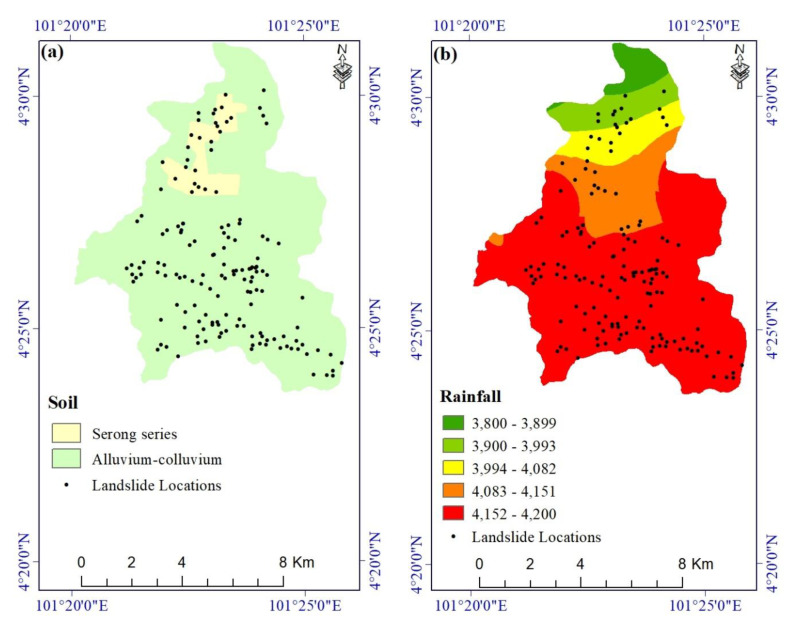
Landslide conditioning factors: (**a**) soil and (**b**) rainfall.

Many researchers consider soil to be an important contributor to slope failures [68,73,74]. Whether a landslide is shallow or deep-seated depends greatly on the Earth materials and the thickness of soil on a slope [75,76]. In this study, the soil layer was digitized from a soil map acquired from the Malaysia Department of Agriculture. In the study area, there are two different groups of soil, namely the Serong Series and soils on alluvium and colluvium (Figure 8a).

A rainfall map of the study area (Figure 8b) was extracted from the TRMM dataset. Natural vegetation cover is conditioned by precipitation and temperature, and in turn, it affects evapotranspiration, rainfall interception, infiltration, and soil characteristics [68,77].

Before we could proceed with the landslide modeling, we defined classes for each of the conditioning factors using ArcGIS. To do this, we first considered potential classes for our conditioning factors based on previous work [78,79,80,81]. Then, we established classes to capture the ranges of factor values characteristic of our study area [29,82,83].

### 3.2. Methods Used

#### 3.2.1. One Rule (One-R) Feature Selection Technique

We used the One-R feature selection technique to measure the effectiveness of each conditioning factor for landslide prediction, as it is a straightforward and effective method for evaluating features based on error rates [79]. In this algorithm, the weight (average merit (AM)) for each factor was obtained based on a few rules and computing error ratios. One-R boosts the quality of input data, leading to more precise modeling output.

#### 3.2.2. Altering Decision Tree (ADTree)

ADTree combines a decision tree with a boosting algorithm [72,84] to increase the prediction quality in binary classification modeling [47,85]. The decision tree in the ADTree model is grown using a boosting algorithm for numeric prediction, in which a decision node and its two prediction nodes are constructed at each boosting iteration step [37,47]. The contribution of the node to the final prediction is computed by a weight that is assigned to each of the prediction nodes. The final prediction probability is based on the summation of all the weighted nodes. This procedure differs from other decision tree-based classifiers such as C4.5 or classification and regression tree (CART), in which a sample follows only one path through the tree [24]. In this study, we tuned the parameters with a trial-and-error procedure: debug = false, number of boosting iterations = 10; random seed = 1, and search path = expand all paths.

#### 3.2.3. AdaBoost (AB)

AdaBoost is an ensemble learning technique proposed by Freund and Shapire [86]. It constructs a strong classifier from a set of weak classifiers and reduces the sensitivity to noisy data. It assigns a weight to each parameter in the training dataset in a repetitive manner. The process is terminated when the pre-defined stopping criteria (e.g., lowest error) are reached [87]. AB works on an adaptive re-sampling technique as follows: (a) a training subset, the data of which are assigned equal weights is randomly generated from the original training dataset; (b) the misclassified cases receive greater weights, whereas the weights of the correctly classified cases remain the same; and (c) the first step is repeated, followed by a normalization process, and a new training subset is generated. AB has several parameters that must be tuned for the best performance. In this study, we tuned the parameters using a trial-and-error process: debug = false, number of boosting iterations = 15, number of seeds = 3, and weight threshold = 100.

#### 3.2.4. Ensemble AB-ADTree Model

**Figure 9:**
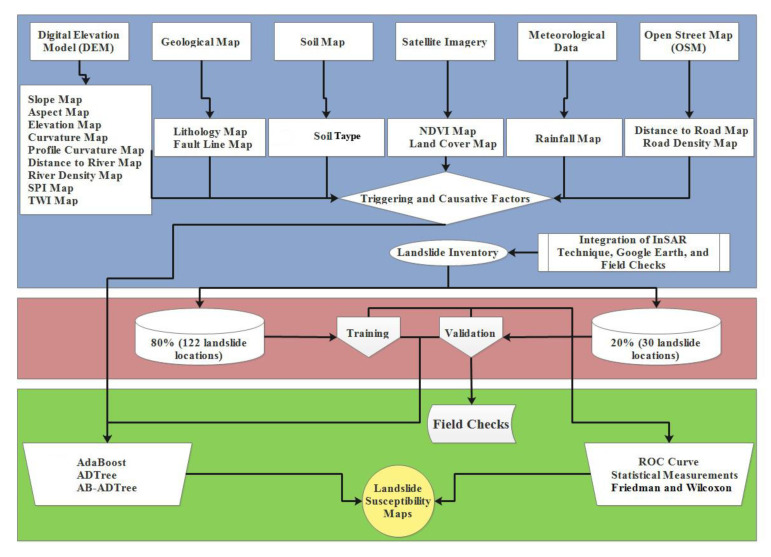
Research methodology for landslide susceptibility mapping in the Cameron Highlands, Malaysia.

In this study, we combined the AB technique with ADTree to create the AB-ADTree ensemble model. The main four steps in using AB-ADTree for landslide susceptibility modeling areas follows: (1) selection of the most important conditioning factors using the One-R technique, (2) training the AB-ADTree ensemble model, (3) validation and comparison of the models, and (4) development of landslide susceptibility maps (Figure 9).

### 3.3. Comparison and Evaluation Metrics

When a new machine learning method is introduced, its performance must be evaluated quantitatively using a real-world database (in our case, the data are the validation dataset of 30 landslides and 30 non-landslides) to determine its predictive power and applicability [88]. Below, we summarize the comparison and evaluation statistical metrics that we use to accomplish this objective, specifically positive predictive value (PPV), negative predictive value (NPV), sensitivity, specificity, accuracy, root mean square error (RMSE), sensitivity, specificity, accuracy, ROC curve, and the Friedman and Wilcoxon tests.

#### 3.3.1. Statistical Metrics

We computed statistical metrics based on the confusion matrix shown in Table 2. In this matrix, true positive (TP) refers to the number of pixels that are correctly classified as landslide, whereas true negative (TN) is the number of pixels that are correctly classified as non-landslides. False positive (FP) and false negative (FN) are the number of pixels that are incorrectly classified, respectively, as landslides and non-landslides.

Sensitivity, specificity, and accuracy are calculated from the confusion matrices derived from the models as follows:

(1) $$\mathrm{Sensitivity}=\frac{\mathrm{TP}}{\mathrm{TP}+\mathrm{FN}}$$

(2) $$\mathrm{Specificity}=\frac{\mathrm{TN}}{\mathrm{TN}+\mathrm{FP}}$$

(3) $$\mathrm{Accuracy}\;(\mathrm{Efficiency})=\frac{\mathrm{TP}+\mathrm{TN}}{\mathrm{TP}+\mathrm{TN}+\mathrm{FP}+\mathrm{FN}}.$$

Sensitivity is defined as the ratio of correctly classified landslides to all predicted landslides. Specificity is the ratio of incorrectly classified landslides to all predicted non-landslides. Accuracy is the ratio of correctly classified landslide pixels to correctly classified non-landslides pixels [89].

In addition, we computed root mean square error (RMSE) (Equation (4)), which is a measure of the size of the error between the model outputs and observations. The smaller RMSE, the higher model performance [89,90,91].

(4) $$\mathrm{RMSE}=\sqrt{\frac{1}{n}}\sum_{i=1}^{n}(X_{\mathrm{predicted}}-X_{\mathrm{actual}}{)}^{2}$$

where n is the number of values in the training dataset, ‘X predicted’ are the predicted values in the training dataset, and ‘X actual’ are the observed values.

#### 3.3.2. Receiver Operating Characteristics (ROC) Curve

The ROC curve is a widely used method for evaluating the performance of empirical learning systems. The graphical plot of the ROC curve includes a sensitivity y-axis and a false-positive rate x-axis (1-specificty). The ROC curve can be used in conjunction with machine learning methods to evaluate the performance of a classifier [92]. Performance is quantitatively defined using the area under the ROC curve (AUC) [93,94]. An optimal classifier has an AUC value equal to 1, whereas the AUC value of a random classifier is ≤0.5 [95,96].

#### 3.3.3. Friedman and Wilcoxon Tests

We employed the Friedman and Wilcoxon tests to compare the predictive capabilities of the models used in this study. The Freidman test shows overall statistical differences between the models and is used for two-way analysis of variance of non-parametric data [97]. The Wilcoxon test [98] is used for comparing the performance of two or more samples from the same community [33]. The tests are judged based on two possible hypotheses [9]: first, there is no significant difference between the predictive capabilities of the models (H0); second, there is a statistical difference between the predictive capabilities of the models (H1). The Friedman test judges whether there is a statistical difference between two models if the H0 hypothesis is true (*p*-value < 0.05), whereas the Wilcoxon test determines *p*- and *z*-values to perform a pairwise test between the models. The models are statistically different if the *p*-value < 0.05 and if +1.96 > *z*-value > −1.96 [34,93,99].

## 4. Results

### 4.1. Factor Importance

**Figure 10:**
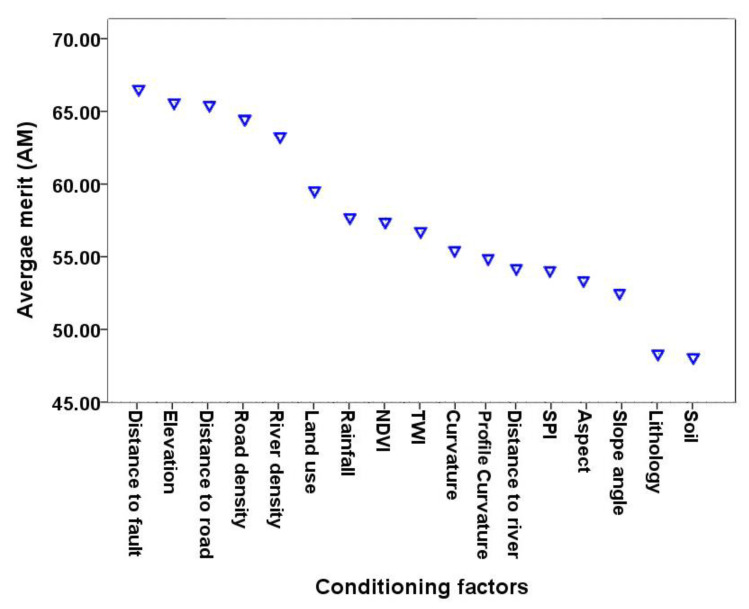
Factor importance measured using the One-R method.

The prediction capability (merit) of the conditioning factors used in this study is shown in Figure 10. The results, which were obtained using the One-R technique with 10-fold cross-validation, indicate that the distance to fault has the highest merit (66.529) among the landslide conditioning factors, followed by elevation (65.290), distance to road (65.428), road density (64.463), river density (63.270), land use (59.551), rainfall (57.576), NDVI (57.393), TWI (56.750), curvature (55.372), profile curvature (55.001), distance to river (54.821), SPI (54.132), aspect (53.994), slope angle (53.304), lithology (48.705), and soil (47.705).

### 4.2. Performance Analysis

Results of the goodness-of-fit and prediction accuracy of the models based on the training and validation datasets, respectively, are shown in Table 3. For the training dataset, the sensitivity, specificity, accuracy, and RMSE of the ADTree algorithm are, respectively, 79.5%, 75%, 77%, and 0.443. Corresponding values for AB are 86.9%, 84.4%, 85.7%, and 0.301, and those for the AB-ADTree ensemble algorithm are 83.6%, 82%, 82.8%, and 0.315. In the case of the validation dataset, the AB ensemble model has higher sensitivity (86.2%), specificity (83.9%), and accuracy (85%), and a lower RMSE (0.212) than the AB-ADTree (79.3%, 77.4%, 78.3%, and 0.289) and ADTree (76.9%, 70.6%, 73.3%, and 0.366) models. Based on these performance metrics, we conclude that the AB ensemble model is more accurate than the AB-ADTree and ADTree models in predicting landslide susceptibility in our study area.

### 4.3. Landslide Susceptibility Maps

After the modeling process and selecting the most reasonable results based on the parameter tuning of each model, we ran the ADTree, AB, and AB-ADTree algorithms on the training dataset. We calculated landslide susceptibility indexes (ISIs) based on the probability distribution functions of the algorithms and prepared landslide susceptibility maps based on these indexes as follows:

#### 4.3.1. ADTree Landslide Susceptibility Map

**Figure 11:**
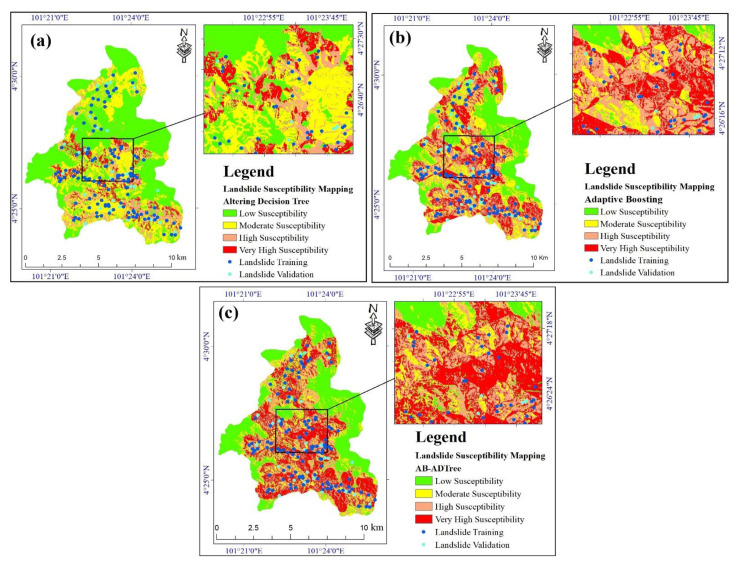
Landslide susceptibility maps: (**a**) ADTree; (**b**) AB; and (**c**) AB-ADTree.

The validation results showed that the ADTree model performed very poorly, indicating that this model is unsuitable for landslide susceptibility mapping in our study area. However, we still produced a landslide susceptibility map with four susceptibility classes (low, moderate, high, and very high susceptibility) using the ADTree model (Figure 11a).

The low susceptibility class covers about half of the study area (40.459 km^2^), in comparison to the very high susceptibility class, which covers only 6% of the area (5.220 km^2^). The areas of the moderate and the high susceptibility classes are 32% (25.634 km^2^) and 12% (9.935 km^2^), respectively.

#### 4.3.2. AB landslide Susceptibility Map

The AB model assigned 29% (23.264 km^2^) of the study area in the high susceptibility class; values for the low, moderate, and very high susceptibility classes are 28% (22.934 km^2^), 22% (18.081 km^2^), and 21% (16.968 km^2^), respectively (Figure 11b).

#### 4.3.3. AB-ADTree Landslide Susceptibility Map

The ensemble AB-ADTree model performed better than the ADTree model, but it was outperformed by the AB model. The high and very high susceptibility classes cover 53% (42.729 km^2^) of the study area (Figure 11c). The low and moderate susceptibility classes have areas of 31% (25.229 km^2^) and 16% (13.282 km^2^), respectively.

#### 4.3.4. Validation and Comparison of Landslide Susceptibility Maps

**Figure 12:**
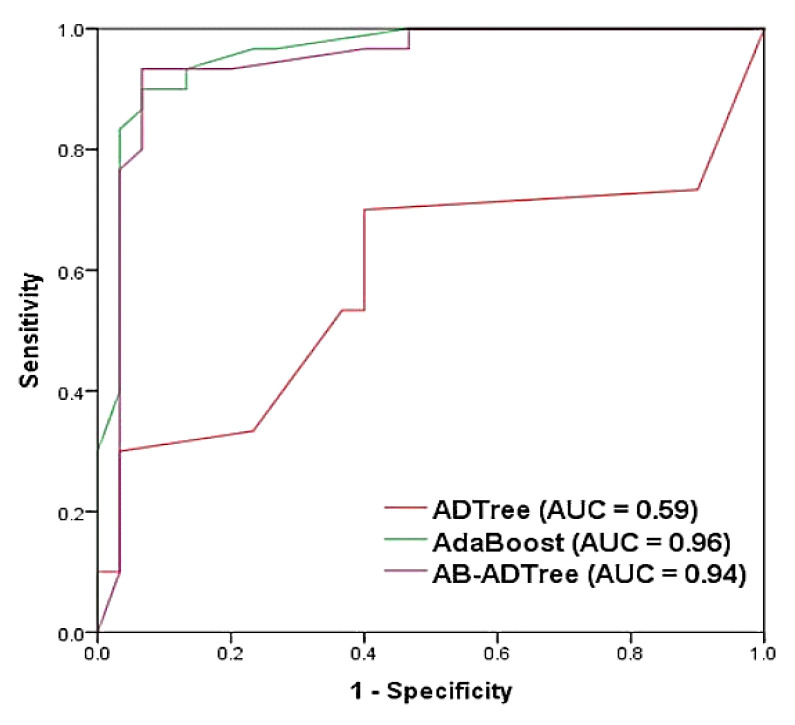
Receiver Operating Characteristic (ROC) curve and area under the receiver operating characteristic curve (AUC) values of the models using validation dataset.

We used the AUC metric to determine the prediction accuracy of the models. Figure 12 shows the ROC curves and related AUC values for the three models. The AB and AB-ADTree models have similar performances, with AUC values of 0.96 and 0.94, respectively. In contrast, the ADTree model, with an AUC value of 0.59, performed poorly as a landslide predictive model.

Table 4 shows the results of the Friedman test. The mean ranks for the AB, AB-ADTree, and ADTree models are, respectively, 2.72, 2.20, and 1.08. The results indicate that there is a large difference between ADTree and the other two models in terms of their abilities to predict future landslides. Further, the high chi-square value (83.633) and low significance (0.000) of AB suggest that there is a large difference among the models. When one of the tested models has a low mean rank (in this case ADTree), the Friedman test assigns a high chi-square value and a low significance to one of the other models to indicate that there is a large difference among them.

The Wilcoxon test was used to assess pairwise differences among the models. Table 5 shows there are statistical differences between ADTree and the other two models, with a *p*-value of 0.000 and *z*-value of −6.737 when compared to AB, and −6.472 and 0.000 when compared to AB-ADTree. The results also show that there is a significant difference between the AB and AB-ADTree models (*p*-value = 0.041).

## 5. Discussion

All 17 landslide conditioning factors used in this study are deemed to be important, because they have positive values of average merit based on the One-R technique. We found that fault distance is the most important conditioning factor for landslide occurrence in our study area, suggesting that the nearer a location is to a fault, the higher the probability of landslide occurrence. Fault movements deform and fracture rock, decreasing its strength and facilitating landslides on steep slopes along roads, rivers, and streams [100,101]. Although the effect of fault distance cannot be directly analyzed and observed through field surveys, our results indicate that it is an important factor. This finding is not in accord with the relation between fault distance and landslide occurrence reported by Cevik and Topal [102], but many other researchers have argued that fault distance is one of the most important factors for landslide occurrence worldwide [41,103,104,105,106]. Field observations confirmed our modeling results that anthropogenic factors such as road construction and hydrology factors such as rainfall have a significant role in landslide occurrence in the study area.

Landslide researchers have applied a variety of machine learning approaches to different regions and have achieved different results. Even within a single region, different models, such as logistic regression and support vector machine, may yield different results due to weighting differences, which, in turn, relate to their probability distribution functions. These differences stem, in part, from epistemic uncertainties in model selection and input data. A consequence of the different methods used during the modeling process is that there is no agreed-upon framework for landslide susceptibility mapping. To reduce epistemic uncertainty, we require comprehensive trial-and-error studies of landslide conditioning factors and landslide susceptibility mapping methods. Newer machine learning models have overcome the over-fitting and noise challenges that previously arose during the modeling process, and their goodness-of-fit and performance have improved in comparison to more conventional models [32,33,61,62,107]. Recently, researchers have developed promising new ensemble models that are more powerful than individual models [105]. In this study, we improved and enhanced the ADTree algorithm by creating, using, and testing the ensemble AB-ADTree model. We show that this model provided higher prediction accuracy than the ADTree as an individual algorithm.

The performance of the machine learning models used in this study was evaluated using statistical parametric and non-parametric methods. The results show that outperformed the AB-ADTree and ADTree models. The new model successfully distinguished landslide-prone areas in the study area based on the training and validation datasets. Our findings support previous studies that indicated that the AB ensemble technique and its derived ensemble models can significantly decrease over-fitting and the noise problems of the modeling process [55,87,88,90,105,108,109].

There are several published papers that report on the capability of the AB ensemble technique for improving the performance of the base models. Hong et al. [72] achieved promising results by combining the AB ensemble technique with J48 to predict landslides in the Guangchang area, China; and Bui et al. [110] improved the predictive performance of the functional tree model using the AB ensemble technique to predict landslides along a national road in Vietnam. Abedini et al. [43] combined the Bayesian logistic regression (BLR) with the AB ensemble technique and reported on the improved prediction accuracy for landslide susceptibility in Kamyaran, Iran. Wu et al. [24] improved the capability of the ADTree model with the AB ensemble technique in a study of landslides in Longxian County, China. Finally, in a recent study, Tran et al. [32] showed that the AB ensemble model performed better than Bagging, Dagging, Decorate, and Real AdaBoost for improving the performance of the Hyperpipes algorithm in predicting landslide susceptibility in the Nam Dam Commune, Vietnam.

Researchers have also used this modeling approach in flood and gully erosion prediction and groundwater potential mapping. In a recent study, Pham et al. [111] combined the AB ensemble technique with the Credal decision tree to predict floods in the Markazi Province of Iran. They showed that this technique performed better than Bagging, Dagging, and MultiBoost. Nhu et al. [55] coupled a reduced pruning error tree model with AB, Bagging, and Random Subspace techniques for gully erosion susceptibility mapping using in the Shoor River watershed of Iran. Nguyen et al. [61,62] proposed ensemble modeling based on the ANN and logistic regression for groundwater potential mapping in two different regions of Vietnam.

## 6. Conclusions

Landslides are common in the Cameron Highlands, Malaysia, and they cause much damage to roads, buildings, and other infrastructure. Losses are likely to increase in the future due to increased urbanization and land clearing. Local governments, as well as the Malaysian federal government, are concerned about the possibility of loss of life due to landslides, especially during heavy rainfall, which is common in the country. To manage this problem, Malaysian policy- and decision-makers require a better understanding of where landslides are likely to occur. Accurate landslide susceptibility maps help them select suitable locations for infrastructure development.

We employed the InSAR technique, Google Earth images, and field investigation to inventory landslides in our study area. From a dataset of 152 landslides; 20% (30 landslides) were used for validation purposes, and the remainder (122 landslides) were used to train ADTree, AB, and AB-ADTree machine learning algorithms. The 17 landslide conditioning factors (slope, aspect, elevation, distance to road, distance to river, proximity to fault, road density, river density, NDVI, rainfall, land cover, lithology, soil types, curvature, profile curvature, SPI, and TWI) used in this study were obtained from a variety of sources, including a DEM, geological map, soil map, the Tropical Rainfall Measuring Mission sensor, satellite imagery, and Open Street Map. We created landslide susceptibility maps using the ADTree, AB, and AB-ADTree algorithms, and we validated the models using AUC and the statistical metrics PPV, NPV, sensitivity, specificity, accuracy, and RMSE. The ADTree, AB, and AB-ADTree models have AUC values of, respectively, 59%, 96%, and 94%. The Friedman and Wilcoxon statistical tests were used to assess model performance. These tests showed that the ADTree model performed much more poorly than the other two models. Further, the single AB model performed better than the ensemble AB-ADTree model in predicting landslide susceptibility in the study area. This study provides insights into the development of more efficient and accurate landslide predictive models that can be used to mitigate landslide hazards.

## Figures and Tables

**Table 1:** Source and scales for the landslide conditioning factors used in this study. DEM: Digital Elevation Model, NDVI: Normalized Difference Vegetation Index, SPI: Stream Power Index, TRMM: Tropical Rainfall Measuring Mission, TWI: Topographic Wetness Index.

Conditioning Factor	Source	Scale	Classification Method
Slope angle	DEM generated from Sentinel-1 satellite imagery	10 × 10 m	Manual
Aspect			Manual
Elevation (m)			Equal interval
Distance to river (m)			Manual
River density (km/km^2^)			Natural breaks
Curvature			Manual
Profile curvature			Manual
SPI			Natural breaks
TWI			Natural breaks
Lithology	Mineral and Geoscience Department, Malaysia	1:100,000	Lithological units
Distance to fault (m)			Natural breaks
Soil layer	Department of Agriculture, Malaysia	1:100,000	Natural breaks
NDVI	Sentinel-2	10 × 10 m	Natural breaks
Land cover	Sentinel-1 and Landsat-8 images	10 × 10 m	Land cover unit
Rainfall (mm)	TRMM data	10 × 10 m	Natural breaks
Distance to road (m)	Open street map		Natural breaks
Road density (km/km^2^)			Natural breaks

**Table 2:** Technical attributes of the confusion matrix.

		Predicted		
		${X^{\prime }}_{1}$(landslide)	${X^{\prime }}_{0}$(non-landslide)	Sum
**Observed**	${X^{\prime }}_{1}$(landslide)	TP	FN	P
	${X^{\prime }}_{0}$(non-landslide)	FP	TN	N

**Table 3:** Goodness-of-fit and prediction accuracy of the models for the training and validation datasets.

Factors	ADTree		AB		AB-ADTree	
	T _🞶_	V _🞶_	T	V	T	V
TP	89	20	106	25	102	23
TN	99	24	103	26	100	24
FP	33	10	19	5	22	7
FN	23	6	16	4	20	6
Sensitivity (%)	79.5	76.9	86.9	86.2	83.6	79.3
Specificity (%)	75.0	70.6	84.4	83.9	82.0	77.4
Accuracy (%)	77.0	73.3	85.7	85.0	82.8	78.3
RMSE	0.443	0.366	0.301	0.212	0.315	0.289

T _🞶_: training dataset, V _🞶_: validation dataset, AB: AdaBoost, ADTree: alternating decision tree, AB-ADTree: a combination of AdaBoost and alternating decision tree, TP: true positive, TN: true negative, FP: false positive, FN: false negative, RMSE: root mean square error.

**Table 4:** Friedman test statistics results for this study.

Models	Mean Ranks	Chi-Square	Significance
ADTree	1.08	83.633	0.000
AB	2.72		
AB-ADTree	2.20		

**Table 5:** Wilcoxon signed ranks test statistics.

	AB vs. ADTree	AB-ADTree vs. ADTree	AB-ADTree vs. AB
*z* value	−6.737	−6.472	−2.084
*p* value	0.000	0.000	0.041
